# Sex Differences in Blood–Brain Barrier Transport of Psychotropic Drugs

**DOI:** 10.3389/fnbeh.2022.844916

**Published:** 2022-05-23

**Authors:** Christina Dalla, Pavlina Pavlidi, Danai-Georgia Sakelliadou, Tatiana Grammatikopoulou, Nikolaos Kokras

**Affiliations:** ^1^Department of Pharmacology, Medical School, National and Kapodistrian University of Athens, Athens, Greece; ^2^First Department of Psychiatry, Eginition Hospital, Medical School, National and Kapodistrian University of Athens, Athens, Greece

**Keywords:** sex differences, blood–brain barrier, psychotropics drugs, transporters and channels, brain, females, transporters, mental disorders

## Abstract

Treatment of neuropsychiatric disorders relies on the effective delivery of therapeutic molecules to the target organ, the brain. The blood–brain barrier (BBB) hinders such delivery and proteins acting as transporters actively regulate the influx and importantly the efflux of both endo- and xeno-biotics (including medicines). Neuropsychiatric disorders are also characterized by important sex differences, and accumulating evidence supports sex differences in the pharmacokinetics and pharmacodynamics of many drugs that act on the brain. In this minireview we gather preclinical and clinical findings on how sex and sex hormones can influence the activity of those BBB transporter systems and affect the brain pharmacokinetics of psychotropic medicines. It emerges that it is not well understood which psychotropics are substrates for each of the many and not well-studied brain transporters. Indeed, most evidence originates from studies performed in peripheral tissues, such as the liver and the kidneys. None withstanding, accumulated evidence supports the existence of several sex differences in expression and activity of transport proteins, and a further modulating role of gonadal hormones. It is proposed that a closer study of sex differences in the active influx and efflux of psychotropics from the brain may provide a better understanding of sex-dependent brain pharmacokinetics and pharmacodynamics of psychotropic medicines.

## Introduction

**FIGURE 1:**
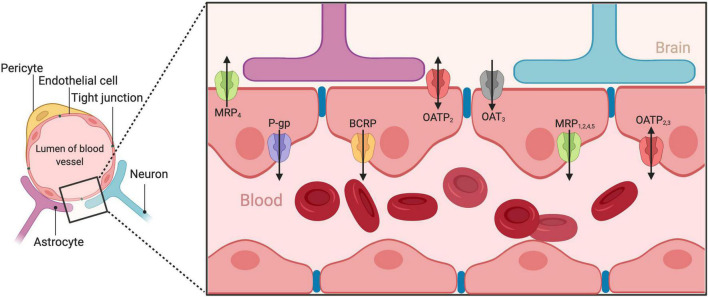
Graphical representation of the blood–brain barrier and its transporters.

Neuropsychiatric disorders carry a significant burden and disproportionally affect more women than men (Wittchen et al., 2011). Their treatment relies on effective drug delivery to the brain. However, such drug delivery is challenging, as the blood–brain barrier (BBB) allows only endo- and xeno-biotics (including medicines) with specific physicochemical characteristics (lipophilicity, molecular weight, and charge) to enter. This barrier is achieved as brain capillary endothelial cells (BCECs), in very close proximity between them, form complex and tight junctions (Figure 1). The BBB functions within the context of the neurovascular unit (McConnell et al., 2017), a structure consisting of neurons, interneurons, astrocytes, pericytes, basal lamina covered with smooth muscular cells, microglia as well as endothelial cells and extracellular matrix, and regulates the cerebral blood flow (Muoio et al., 2014). Although some substances may diffuse passively though the BBB, the influx and efflux of most substances is actively regulated by a complex system of transporters expressed on the BBB. Emerging evidence suggests that brain pharmacokinetics, and thus psychotropic pharmacodynamics is greatly influenced by these transport systems (O’Brien et al., 2012). However, such knowledge is relatively new and now unfolding for many of those systems, especially with the help of evidence gathered from the presence of those transporters in peripheral barriers, such as in the gastrointestinal tract, the liver, and the kidneys. On the other hand, there is strong evidence that many neuropsychiatric disorders present significant sex differences (Balta et al., 2019) and preclinical research is progressing into incorporating sex as an important biological variable (Butlen-Ducuing et al., 2021). Moreover, psychotropic medication present noteworthy pharmacodynamic and interestingly, pharmacokinetic sex differences (Kokras et al., 2011; Seeman, 2021). Given that psychotropic medication must reach the brain to exert their therapeutic action, it emerges that potential sex differences in the brain’s transport systems might be involved in the action of psychotropic medicines in men and women. Therefore, in this minireview, we gather preclinical and clinical findings on how sex and sex hormones can influence the activity of BBB transporters and, discuss the current state of the art.

## P-Glycoprotein

The ABCB1 gene expresses P-glycoprotein (P-gp) (or multi-drug resistance protein 1) in humans and two homologs in rodents, the abcb1a and abcb1b (O’Brien et al., 2012). P-gp has a broad binding site for a wide range of substances, as it is not restricted stereochemically and currently is the most studied transport protein. Regarding psychiatric disorders, P-gp plays an important role in CNS drugs bioavailability (De Klerk et al., 2011). Several antidepressants, like citalopram/escitalopram, paroxetine, imipramine, and venlafaxine are substrates of P-gp (Uhr and Grauer, 2003; Karlsson et al., 2010; O’Brien et al., 2013a,b). Thus, their brain pharmacokinetics are altered by P-gp and response to treatment is affected (Lin et al., 2011). However, other drugs appear not affected by P-gp, like fluoxetine and mirtazapine (Uhr et al., 2000, 2003). Interestingly, some psychotropic medications show a complex interaction with P-gp. For example, sertraline displays a biphasic and time-dependent interaction, fluctuating between inhibition and stimulation of P-gp (Kapoor et al., 2013). Another example is that high doses of nortriptyline saturate the P-gp-dependent transport and thus decrease its clearing effectiveness (Ejsing and Linnet, 2005). Abundant evidence indicates sex differences in the P-gp transport (Baris et al., 2006; Lifschitz et al., 2006; Ueno and Sato, 2012; Tornatore et al., 2013). However, there are also reports showing no significant sex differences (Dagenais et al., 2001; Gottschalk et al., 2011; Long et al., 2016). Such discrepancies, as discussed later, are probably explained by several factors, such as differences in species, the studied substrate, the tissue sampled, etc. Moreover, many P-gp polymorphisms affecting therapeutic drug efficacy are reported (Dizdarevic et al., 2014; Peng et al., 2015; Skalski et al., 2017; Rahikainen et al., 2018). Some are linked with sex-differentiated drug responses and development of specific side effects (Alzoubi et al., 2013; Rahikainen et al., 2018). This highlights the importance of sex segregation in pharmacogenetic research. Lastly, there is evidence that gonadal hormones, such as estrogens, testosterone and progesterone affect the activity of P-gp, and its activity may vary across the menstrual cycle (Axiotis et al., 1991; Peng et al., 2015; Kanado et al., 2019).

## Breast Cancer Resistant Protein

Breast cancer resistant protein (BCRP) is an ABC transporter expressed in different tissues, including the brain epithelial cells, and may be responsible for the low bioavailability of several psychotropics. A recent study showed that sertraline is a BCRP substrate along with its P-gp inhibiting properties (Feng et al., 2019). Venlafaxine dose-dependently induces the BCRP expression (Bachmeier et al., 2011). Moreover, BCRP is known to work in synergy with P-gp, cooperatively eliminating xenobiotics from the brain and thus impeding treatment (Kodaira et al., 2010; Agarwal et al., 2011). Several preclinical studies highlight sex differences in BCRP, whose regulation is testosterone-induced and estradiol-inhibited, and point to a higher expression of BCRP in males (Teo et al., 1993; Fu et al., 2012). Hormonal manipulations, such as gonadectomy or hormonal treatment significantly affected its expression, and in general lower BCRP expression in females led to higher drug exposure (Merino et al., 2005; Gulilat et al., 2020). However, most results are obtained from tissues other than the brain. Interestingly a single study showed that specifically in the brain, BCRP expression is higher in female than male mice (Tanaka et al., 2005).

## Multidrug Resistance-Associated Proteins

Multidrug resistance-associated protein (MRP) is a family of ABC transporters comprising of currently seven known members which are located at luminal membranes, and also found at the BBB (Ueno et al., 2010). Although considered to be an important drug transport mechanism, there is limited information regarding most psychotropics. One study showed that phenytoin and carbamazepine brain levels were lower following upregulation of MRP1 (Chen et al., 2013). Interestingly, no sex differences were identified regarding Mrp1 and Mrp2 mRNA expression in the choroid plexus. However, after its removal, BBB expression levels of Mrp1, Mrp2, and Mrp4 were twice as higher in female mice than in males (Flores et al., 2017). Studies in tissues such as the liver and the kidneys generally corroborate that females have higher MRP expression (Maher et al., 2005; Lu and Klaassen, 2008) and some evidence points to a progesterone and/or dehydroepiandrosterone regulation of this sex difference (Rost et al., 2005; Evseenko et al., 2007).

## Organic Anion Transporters

Organic anion transporter (OAT) is an heterogenous family of negatively charged proteins, mainly located in kidneys and the liver, but OAT1/OAT3 are also found in the brain and are responsible for transporting hydrophobic organic anions. Evidence suggests that valproate, used as a mood stabilizer, is a substrate of OAT1 and homovanillic acid, a metabolite of dopamine, is a substrate of OAT3 (Sekine et al., 2000; Mori et al., 2003). In the kidneys and the liver, OAT expression is affected by androgens, and perhaps different OAT isoforms are stronger expressed in males and females in these tissues. Overall, renal Oat1 expression is androgen-regulated, renal Oat2 expression is modulated by female GH secretion pattern, and hepatic Oat3 expression is influenced by both androgens and female GH secretion pattern (Buist et al., 2003). Although OAT sex differences have been demonstrated in rodents, the direction of sex difference is not consistent and are not confirmed in other species, such as in rabbits (Groves et al., 2006) and in human cells (Breljak et al., 2016). Moreover, regarding specifically the brain, an *in vivo* BBB preclinical study did not identify a sex difference in OAT3 (Ohtsuki et al., 2005).

## Organic Anion Transporting Polypeptides

These transporters form a superfamily of membrane-solute carriers characterized by significant functional diversity and a widespread role in the transport of endo/xenobiotics (Hagenbuch and Meier, 2004). There is scarce data on whether they are involved in the brain transport of psychotropics, but we know that transport of DHEA-S and opioids occurs *via* OATP1A2 and a small sex difference favoring women was recently reported (Asaba et al., 2000; Gao et al., 2000; Taniguchi et al., 2020). However, DHEA administration led to a gender-neutral Oatp1a1 and Oatp1b2 decrease and a further decrease in Oatp1a4 expression only in males (Rost et al., 2005). Evidence on sex differences is convoluted because there are many organic anion transporting polypeptide (OATP) transporters with a broad tissue distribution. Most preclinical evidence converges that activity of Oatp1a4, which is also expressed in the BBB, is higher in females, with testosterone probably suppressing it (Zhang et al., 2013; Brzica et al., 2018). However, several preclinical studies showed a tissue-specific variability in the direction or even absence of sex differences regarding various members of the OATP family (Cheng et al., 2005, 2006; Fu et al., 2012; Muzzio et al., 2014; Prasad et al., 2014; Badee et al., 2015).

## Organic Cation Transporters

Organic cation transporter (OCT) are responsible for transporting cationic substances, like monoamine neurotransmitters, nicotine, the opioid agonist oxycodone, and antipsychotics like amisulpride and haloperidol (Bostrom et al., 2006; Okura et al., 2008; Sekhar et al., 2019). Interestingly, OCT2 and rOCT are found in the brain, and regulate the concentration of neurotransmitters in the neurons rather than the BBB (Busch et al., 1998). Very few data exist on potential sex differences, mostly on renal OCT2, which is expressed more strongly in males than females and it is upregulated by androgens (Alnouti et al., 2006; Groves et al., 2006; Basit et al., 2019). Plasma membrane monoamine transporter (PMAT/SLC29A4), a known transporter for cationic substances, is implicated in the efflux of amisulpride and haloperidol from the brain and is inhibited by nicotine (Tega et al., 2018; Sekhar et al., 2019). Some evidence on sex differences exist for PMAT, as behavioral changes were noted only in female, but not male, PMAT knockout mice (Gilman et al., 2018).

## Monocarboxylate Transporters

Monocarboxylate transporter (MCT) mediate the transport of short chain monocarboxylates such as lactate and pyruvate, indicating their involvement in regulating brain energy substrates. Of 14 MCT members identified, MCT1, MCT2, MCT4, and the sodium-coupled SMCT1 have been described in the brain (Pierre and Pellerin, 2005). They are implicated in the brain transport of several drugs, including notably statins, salicylates and in relation to psychotropics, valproic acid, and γ-hydroxybutyrate (GHB) (Vijay and Morris, 2014). Sex differences have been identified, and are attributed in a tissue-specific regulation by both male and female sex hormones (Felmlee et al., 2020). Hepatic MCT1 and MCT4 regulation appears dependent on both estrogens and androgens (Cao et al., 2017). In muscles testosterone increases MCT1/4 expression but decreases testicular MCT2/4. However, there is a paucity of data regarding sex-dependent patterns of brain MCT regulation, which is important given the tissue-specific profile that emerges.

## Multidrug and Toxin Extrusion Proteins

Multidrug and toxin extrusion protein (MATE) family transporters function in concert with OCT, are mostly expressed in the liver and the kidneys, but they are also found in the brain, and are involved in the transport of cationic drugs (Lickteig et al., 2008). Amisulpride and haloperidol, both antipsychotics, as well as nicotine, have been identified as possible substrates of MATE1 (Tsuda et al., 2007; Sekhar et al., 2019). This family of transporters is very recently discovered, and few data exist on potential sex differences. No data is available for the brain, but it appears that hepatic mRNA of MATE1 was notably increased in females in relation to males, but on the contrary, renal mRNA expression was found notably lower in females compared to males (Lickteig et al., 2008; Fu et al., 2012).

## Other Transporters

Several other transporters, of which relatively little is known, are located at the BBB. Alanine/serine/cysteine transporter 2 (ASCT2) is located at the abluminal membrane of BACEs and is the only transporter of the Solute Carrier 1A (SLC1A) family to transport glutamine (Albrecht and Zielinska, 2019). BBB also expresses Betaine/GABA transporter-1, which in mice can be found as GAT2 transporter, regulating the efflux of GABA, and is different from GABA transporters, GAT1/3, that mediate transport across neurons and astrocytes (Takanaga et al., 2001). Enkephalins and AVP are effluxed by Peptide Transport System 1 and 2, respectively (Banks, 2006; Ueno et al., 2010). Several sodium-coupled transporters (NHE1, NHES, NBCn1, and NKCC1) are implicated in the active transport of lithium, a mood stabilizer across the BBB (Luo et al., 2018). System A and System L are transport systems of small and large neutral amino acids, respectively. Several drugs are carried by system L into the brain, and there is a strategy to design drugs that resemble the amino acids L-histidine and L-tryptophan for enhanced CNS delivery through LAT1 transporter (Hall et al., 2019). However, for all those transport systems little is known about their potential sex differences.

## Discussion

In this minireview we summarized findings about sex differences in brain transport systems. These may affect pharmacokinetics of psychotropic medications in a sex-dependent manner and are important for precision medicine and treatment. In summary, for many transporter systems little is known about their function and the role of sex and gonadal hormones. Some protein transporters are indeed recently discovered, but for many other, evidence accumulates at a slow pace. Moreover, data are more abundant for the peripheral expression and function of these transporters, and less is known about the BBB, with the exception perhaps of the P-gp. This is surprising, as brain-transport systems regulate the influx and massively the efflux (clearance) of psychotropics. Moreover, BBB dysfunction has been implicated in many neuropsychiatric disorders and other diseases which are sex-differentiated (Greene et al., 2020; Profaci et al., 2020; Dion-Albert et al., 2022b). Admittedly, studies on peripheral transporters are methodologically easier, especially in humans where access to the BBB is significantly hindered. However, preclinical studies are also lacking, and more research is needed on which psychotropics are substrates of which BBB transporter system and whether this is sex-differentiated. This research could lead to clinical important findings regarding the treatment of psychiatric disorders in a more precise way.

Despite the paucity of evidence, preclinical studies collectively support the notion of male and female predominant transporters mainly in the periphery (Maher et al., 2005; Klaassen and Aleksunes, 2010; Zhu et al., 2017; Basit et al., 2019). The existence of protein transporter systems in the periphery also adds another layer of complexity in understanding their impact on pharmacokinetics. Most, if not all, of those transporters are heavily expressed in peripheral tissues (intestine, liver, and kidneys) that are crucially implicated in absorption, distribution, and metabolism of drugs. Peripheral transporters play as much an important role in psychotropic pharmacokinetics as do the BBB transporters in delivering to and clearing psychotropics from the brain. Therefore, a psychotropic that is a substrate for a specific transporter may be more extensively absorbed, more broadly distributed and at the same time more readily cleared from the brain and then metabolized and excreted. It remains unknown whether these effects cancel themselves out and, in the context of this review, whether male or female sex affects those transporters equally, in all of their expression sites (brain and periphery) (Cummins et al., 2002; Gottschalk et al., 2011). It is possible that their function is also influenced locally by estrogens – or other steroid – receptors in the BBB. These local interactions represent an interesting new research pathway that could promote our understanding of the BBB and its transporter proteins in the healthy and diseased brain in a sex-dependent way.

Indeed, transporter function, and thus potential sex differences, are not necessarily identical in peripheral tissues (such as in liver, kidneys, and intestine) and the brain. Although transporters present significant, but not absolute conservation across species, some sex differences observed in one species are not confirmed in another. Therefore, future research should focus on whether findings from one species to another are translatable, regarding both substrates for each transporter, as well as on the significance of potential sex differences of transporters in relation to human disease and treatment. A recent study on P-gp comparing gastro-intestinal tissue from Wistar rats and humans confirmed the translatability of experimental findings on discovered sex differences (Mai et al., 2021). P-gp activity is altered in patients with depression and recent evidence, in post mortem brain, suggest that vascular alterations in the BBB are present in women with depression (de Klerk et al., 2009; Dion-Albert et al., 2022b). Interestingly, BBB dysfunction has been associated with many other diseases, such as dementia, autoimmune disorders, epilepsy, and stroke, that also present sex differences and often co-exist with depression (Greene et al., 2020; Profaci et al., 2020). Therefore, future studies should investigate sex differences in specific transport proteins of the BBB in relation to its dysfunction during depression and other comorbidities. Moreover, transporter activity may be affected by factors such as stress, disease, exercise, or diet in a brain-region specific manner. Indeed, chronic variable stress altered BBB integrity in female, but not in the male mouse prefrontal cortex and this could have contributed to stress vulnerability (Dion-Albert et al., 2022b).

This mini-review focused on sex differences in psychotropic transport across the BBB. As the purpose of such sex differences remains unclear, it is postulated that the mammalian reproductive process exerted a selection pressure that explains those sexual dimorphisms (Gilks et al., 2014; Della Torre and Maggi, 2017). As elegantly reviewed elsewhere, this is reflected to several sex differences at the BBB in health and disease, regarding, but not limited to BBB strength, metabolism, response to stressors and involvement of several pathways, classic and non-classic genomic, as well as non-genomic, involving NO signaling, matrix metalloproteinases, the RhoA/Rho-kinase-2 pathway and other estrogens-mediated pathways (Weber and Clyne, 2021; Dion-Albert et al., 2022a).

In conclusion, accumulated evidence supports the existence of several sex differences in expression and activity of BBB transporters, and a further modulating role of gonadal hormones. A closer study of sex differences in the active influx and efflux of psychotropics from the brain may provide a better understanding of sex-dependent brain pharmacokinetics and pharmacodynamics of psychotropics. This would have a significant impact in precision medicine and treatment. Furthermore, in combination with BBB permeability studies, research on sex differences in BBB transporters will contribute to our understanding of the neurobiology and treatment of psychiatric diseases and their relationship with other disorders, such as autoimmune and neurological.

## Author Contributions

D-GS and TG searched the literature and gathered relevant evidence. PP compiled the first draft. NK and CD conceived and coordinated the study, mastered the final and revised manuscripts, and provided guidance. All authors contributed and approved the manuscript.

## Conflict of Interest

NK and CD have received honoraria and financial support from Janssen-Cilag, Lundbeck, Elpen S.A., and Medochemie S.A. The remaining authors declare that the research was conducted in the absence of any commercial or financial relationships that could be construed as a potential conflict of interest.

## Publisher’s Note

All claims expressed in this article are solely those of the authors and do not necessarily represent those of their affiliated organizations, or those of the publisher, the editors and the reviewers. Any product that may be evaluated in this article, or claim that may be made by its manufacturer, is not guaranteed or endorsed by the publisher.
